# Association between disability status and health care utilisation for common childhood illnesses in 10 countries in sub-Saharan Africa: a cross-sectional study in the Multiple Indicator Cluster Survey

**DOI:** 10.1016/j.eclinm.2023.101870

**Published:** 2023-02-27

**Authors:** Sara Rotenberg, Calum Davey, Emily McFadden

**Affiliations:** a Nuffield Department of Primary Care Health Sciences, University of Oxford, UK; b International Center for Evidence in Disability, London School of Hygiene and Tropical Medicine, UK

**Keywords:** Child illness, Disability, Health equity, Diarrhoeal disease, ARI, Fever

## Abstract

**Background:**

Approximately 70 million children in sub-Saharan Africa (SSA) are disabled, yet little is known about the prevalence of and care-seeking patterns for common childhood illnesses, such as acute respiratory infection (ARI), diarrhoea, and fever.

**Methods:**

Data were from 10 SSA countries with data available from 2017 to 2020 in the UNICEF-supported Multiple Indicator Cluster Survey (MICS) online repository. Children aged 2–4 years who completed the child functioning module were included. Using logistic regression, we examined the association between disability and ARI, diarrhoea and fever in the past two weeks and care-seeking behaviour for these illnesses. Using multinomial logistic regression, we examined the association between disability and the type of health care providers from which caregivers sought care.

**Findings:**

There were 51,901 children included. Overall, there were small absolute differences in illnesses between disabled and non-disabled children. However, there was evidence disabled children had a greater odds of ARI (aOR = 1.33, 95% C.I 1.16–1.52), diarrhoea (aOR = 1.27, 95% C.I. 1.12–1.44), and fever (aOR = 1.19 95% CI 1.06–1.35) compared to non-disabled children. There was no evidence that caregivers of disabled children had a greater odds of seeking care for ARI (aOR = 0.90, 95% C.I 0.69–1.19), diarrhoea (aOR = 1.06, 95% C.I. 0.84–1.34), and fever (aOR = 1.07, 95% C.I 0.88–1.30) compared to caregivers of non-disabled children. Caregivers of disabled children had a higher odds of seeking care from a trained health worker for ARI (aOR = 1.76, 95% C.I. 1.25–2.47) and fever (aOR = 1.49, 95% C.I. 1.03–2.14) or non-health professional (aOR = 1.89, 95% C.I. 1.19–2.98) for ARI than from an unspecified health facility worker compared to caregivers of non-disabled children, but no associations were not seen for diarrhoea.

**Interpretation:**

While the data showed relatively small absolute differences, disability was associated with ARI, diarrhoea and fever and caregivers of disabled children sought care from trained health workers for ARI and fever more than non-disabled children. The overall small absolute differences show closing gaps in illness and access to care may be possible, but highlights that more research on illness severity, care quality, and outcomes should be conducted to further assess health inequities for disabled children.

**Funding:**

SR receives funding from the 10.13039/100016442Rhodes Trust.

**Research in context**

**Evidence before this study**

Globally, UNICEF estimates that there are 240 million disabled children. Many of these children experience exclusion, discrimination, and barriers across all facets of their life—and access to health care is no exception. Disabled children often have higher health needs, worse health outcomes, and face a range of barriers in obtaining high-quality health care. There are limited efforts to monitor progress on disability-inclusion within the Sustainable Development Goals (SDGs), meaning these inequities in child health indicators are often unmeasured. We sought to look at the core prevalence and care-seeking indicators related to childhood illness, as well as trends in where parents sought care for their disabled children. We searched PubMed and Google Scholar using the combination “disability” and “diarrhoea”, “acute respiratory infection”, “fever”, “multiple indicator cluster survey (MICS)”, and “care seeking”. We found one study in the academic literature that examined rates of illness and care-seeking behaviours, but this was only among children ‘at-risk’ of intellectual disability. UNICEF has published global reports disaggregating the MICS data by disability status and individual country reports, but these have not been examined regionally. Furthermore, we found no quantitative studies examining the care-seeking patterns of disabled children.

**Added value of this study**

This multi-country study is the first to compare inequities in child illness indicators for disabled and non-disabled children (as measured by the Washington Group Questions) in sub-Saharan Africa. While the data showed small absolute differences between disabled and non-disabled children, it provides some evidence of higher odds of common childhood illnesses in disabled children and shows that parents of disabled children often seek care for fever and acute respiratory infection (ARI) from qualified health professionals or non-qualified health professional for ARI compared to non-disabled children.

**Implications of all the available evidence**

The study shows a complex picture of health for disabled children. While small absolute differences between disabled and non-disabled children may seem like a positive finding, the small numbers of disabled children and our inability to investigate other factors in this relationship, such as severity of illness, quality of care, and care outcomes, mean that there is more research needed to conclude whether there are disability-based inequities in common childhood illnesses. Our findings of higher odds of common childhood illness among disabled children cannot be due to impairment alone. Instead, a constellation of social and structural factors likely contributes to these relative inequities for disabled children. Efforts to address the social determinants of health within the SDGs should specifically focus on disabled children and their families in order to mitigate inequities. Further, the paucity of data within this study and in the literature highlights the importance of improving disability data collection efforts to monitor disability inclusion for core child health indicators.

## Introduction

There are approximately 70 million disabled children living in sub-Saharan Africa,^1^ though this figure is expected to increase because of population growth, increased childhood survival, conflict, climate change, and inequity on the continent.^2^, ^3^, ^4^ Childhood disability is closely linked with poverty^5^ and health system failures, since limited access to skilled birth attendance and routine immunizations can result in preventable disability.^3^^,^^4^ Improving access to health care services can not only assist in primary prevention of preventable disability, but also better support children with congenital and acquired disabilities who often face barriers in accessing health care.^6^ Limited specialised services, under-trained health workers, poverty and economic barriers, physical inaccessibility, and stigma generally contribute to worse outcomes for disabled children.^7^

The Sustainable Development Goals (SDGs) have renewed focus on decreasing the high burden of neonatal, infant, and child mortality, as well as improving child health overall.^8^^,^^9^ While 80% of under-five mortality still occurs in South Asia and SSA, there are limited data on the health status and outcomes of disabled children in these regions. Round 6 of the UNICEF-supported Multiple-Indicator Cluster Surveys (MICS) is one of the only sources of data both on disability status^10^ and three of the five leading causes of child mortality: acute respiratory infection (ARI), diarrhoeal disease, and fever (used as an indicator of malaria). We examine the odds ratios for having these illnesses, and care-seeking behaviour for them, as reported by caregivers of disabled and non-disabled children. For caregivers who sought care for their child, we also describe the type of health worker they saw to examine whether care-seeking patterns differ between disabled and non-disabled children.

## Methods

### Data

We used individual participant data from MICS6 conducted between 2017 and 2020 for 10 countries in the sub-Saharan African region (Central African Republic, Chad, Democratic Republic of Congo, Gambia, Ghana, Lesotho, Madagascar, Malawi, Sierra Leone, and Togo) that were available as of January 2021. MICS6 data are available for Guinea-Bissau, São Tomé and Príncipe, Zimbabwe, but are not included as there were fewer than 25 disabled children in the denominator for the outcomes of interest.

### Disability measures

The MICS6 surveys use the Washington Group/UNICEF Child Functioning Module (CFM) to measure functional impairment which allows data to be disaggregated by disability status. While the CFM is asked to children 2–17, the overlapping age ranges with outcomes of interest narrowed our population to those aged 2–4. For this age range, the CFM measures impairment across eight functional domains, including vision, hearing, mobility, communication/comprehension, behaviour, learning, dexterity, and playing.^11^^,^^12^ Questions across these domains assess the level of difficulty the individual child has with each task. For example, children are asked “Compared with children of the same age, does (name) have difficulty playing?“, to which they could respond “no difficulty”, “some difficulty”, “a lot of difficulty” or “cannot play at all”. As per the Washington Group guidelines, a child is considered disabled if their caregiver responds with “a lot of difficulty” or “cannot [insert relevant functional domain] at all” in one or more functional domains. Children are considered non-disabled when they answer “no difficulty”, “some difficulty” to all questions. Children with missing data for all CFM questions are not included, while those who answer “a lot of difficulty” or “cannot [insert relevant functional domain] at all” for at least one domain, but have one or more other domains missing, are still included, as per the Washington Group Syntax.^13^ While data may not be missing at random, it was not possible to assess this with the measured variables and our complete case analysis is unlikely to bias the logistic regression estimate.^14^

### Outcome and covariates

For each of the three illnesses (ARI, diarrhoea, and fever), we examined having the illness, whether care was sought, and from which health worker cadre care was sought as our outcomes. Variables for diarrhoea and fever were based on caregiver responses to direct questions on whether or not the child had had symptoms of the respective illnesses in the two weeks prior to the survey interview. ARI was based on caregiver responses to questions on illness with a cough, accompanied by rapid or difficult breathing, and symptoms of a chest problem, with or without congestion. Caregivers were asked whether they sought care for these illnesses and, if they had, to list the source(s) where they sought care. Provider type was grouped into qualified health providers (physicians and public and private community health workers), non-professional providers (relative/friend, shop/market/street, traditional practitioner), and unspecified health facility workers (public or private hospital, health centre, health post, pharmacy, or clinic), based on mapping provider options in the MICS under-five questionnaire to WHO's International Standard Classifications of Occupations (ISCO-08).^15^

Covariates were age, sex, wealth, and location (urban/rural). Age and sex were based on caregiver-reported responses. Location was marked by the interviewer according to how the cluster was designated by UNICEF or country statistical offices. Wealth indices were constructed using household characteristics, including ownerships of goods, living situation, water and sanitation, and other assets (included as a cleaned variable in the data we downloaded from UNICEF).^16^ Given data on co-morbidities, non-standardized BMI scores, and individual-level ethnicity data were not in the dataset, we could not include them as covariates.

### Statistical analysis

Analyses were completed using R 4.1.2 and Stata, version 13 (StataCorp LP, College Station, TX). Baseline summary statistics (means and standard deviations or numbers and proportions) were calculated for covariates and summarised by country, and overall, and by disability status.

Logistic regression was used to examine the relationship between disability status and the having each illness (diarrhoea, fever, and ARI) in the last two weeks, and whether care was sought, in each country and overall.

Multinomial logistic regression was used to examine the relationship between disability status and care provider type for each illness separately. Analyses are presented for all countries combined and by country. All analyses were adjusted for age, sex, location, and wealth; analyses presenting all countries combined were also adjusted for country). All models accounted for the clustered survey design (i.e., country, cluster, household numbers, and sample weights) and used robust standard errors for the confidence interval calculations.

### Ethical approval and reporting guidelines

Publicly available, de-identified data were obtained from UNICEF via their online portal (http://www.mics.unicef.org/). Ethical clearance was the responsibility of the institutions that administered the survey. MICS Data were collected from eligible respondents following informed consent by each national UNICEF-partner institution administering the survey. This study was therefore exempt from University of Oxford ethics review. The funders played no role in the design, collection, analysis, or interpretation of results.

In accordance with journal guidelines, this study conformed to the GATHER statement^17^ for reporting of global health estimates (Supplementary Materials 2).

### Role of funding

The funder had no role in the study design, data collection and analysis, decision to publish, or preparation of the manuscript.

## Results

There were 51,901 children, aged 2–4 years, sampled in the 10 countries in sub-Saharan Africa. Table 1 shows baseline characteristics by country and overall. The number of children sampled ranged from 1335 in Lesotho to 9697 in Chad. The overall prevalence of disability was 7% (n = 3550), and ranged from 4% (n = 247) in Malawi to 11% (n = 423) in Central African Republic. Half the sample were girls (n = 26,013, 50%). The mean age of the sample was 3.4 years old (SD ± 0.6), though this was younger in Sierra Leone (3.0 ± 0.8) and Togo (3.0 ± 0.8). Table 1 also shows the total disability prevalence and prevalence of disability for each functional domain. Learning (42%) and controlling behaviour (38.5%) were the most common functional domains where caregivers reported functional difficulty, while seeing (6.2%) was the least frequently reported type of functional difficulty. These small sample sizes prohibited disaggregation by functional domain.

**Table 1:** Baseline characteristics of 51, 901 children from 10 Multiple Indicator Cluster Survey (MICS) countries in sub-Saharan Africa, 2017–2020.

	All countries combined	Central African Republic (CAR)	Chad	Democratic Republic of Congo (DRC)	Ghana	Lesotho	Madagascar	Malawi	Sierra Leone	The Gambia	Togo
Number of children	51,901	3701	9697	8598	3678	1335	4648	5947	7092	4225	2980
Survey Year	2017–2020	2018–2019	2019	2017–2018	2017–2018	2018	2018	2019–2020	2017	2018	2017
Female (n, %)	26,013 (50%)	1868 (51%)	4801 (50%)	4427 (52%)	1864 (51%)	667 (50%)	2288 (49%)	3031 (51%)	3572 (50%)	2054 (49%)	1441 (48%)
Age (Mean ± SD)	3.40 (0.6)	3.49 (0.5)	3.51 (0.5)	3.48 (0.5)	3.49 (0.5)	3.52 (0.5)	3.50 (0.5)	3.49 (0.5)	3.00 (0.8)	3.48 (0.5)	3.01 (0.8)
Urban (n, %)	13,431 (26%)	1308 (35%)	1713 (18%)	2298 (27%)	1471 (40%)	331 (25%)	1099 (24%)	691 (12%)	2064 (29%)	1541 (37%)	915 (31%)
Wealth quintile (n,%)											
1 (bottom)	15,257 (29%)	1501 (41%)	2352 (24.3%)	2822 (33%)	1128 (31%)	428 (32%)	1464 (32%)	1285 (22%)	1998 (28%)	1539 (36%)	740 (25%)
2	11,462 (22%)	0 (0%)	2313 (23.9%)	2271 (26%)	722 (20%)	317 (24%)	1111 (24%)	1267 (21%)	1771 (25%)	1033 (24%)	657 (22%)
3	10,434 (20%)	732 (20%)	1896 (19.6%)	1788 (21%)	685 (19%)	225 (17%)	910 (20%)	1238 (21%)	1593 (23%)	755 (18%)	612 (21%)
4	8101 (16%)	799 (22%)	1546 (15.9%)	1176 (14%)	583 (16%)	199 (15%)	646 (14%)	1143 (19%)	975 (14%)	520 (12%)	514 (17%)
5 (top)	6647 (13%)	669 (18%)	1590 (16.4%)	541 (6%)	560 (15%)	166 (12%)	517 (11%)	1014 (17%)	755 (11%)	378 (9%)	457 (15%)
Disabled (n, %)	3550 (7%)	423 (11%)	786 (8.1%)	500 (6%)	296 (8%)	89 (7%)	320 (7%)	247 (4%)	451 (6%)	236 (6%)	202 (7%)
Type of impairment											
Seeing	219 (6.2%)	22 (5.1%)	69 (8.8%)	30 (6.0%)	8 (2.7%)	17 (19.1%)	22 (6.9%)	28 (11.3%)	9 (2.0%)	5 (2.1%)	9 (4.5%)
Hearing	306 (8.6%)	41 (9.5%)	148 (18.8%)	38 (7.6%)	6 (2.0%)	7 (7.9%)	14 (4.4%)	21 (8.5%)	12 (2.7%)	11 (4.7%)	8 (4.0%)
Walking	417 (11.7%)	49 (11.3%)	158 (20.1%)	63 (12.6%)	10 (3.4%)	4 (4.5%)	12 (3.8%)	36 (14.6%)	46 (10.2%)	15 (6.4%)	24 (11.9%)
Controlling Behaviour	1368 (38.5%)	157 (36.3%)	231 (29.4%)	186 (37.2%)	179 (60.5%)	58 (65.2%)	98 (30.6%)	109 (44.1%)	96 (21.3%)	154 (65.3%)	100 (49.5%)
Playing	396 (11.2%)	36 (8.3%)	125 (15.9%)	43 (8.6%)	6 (2.0%)	6 (6.7%)	39 (12.2%)	38 (15.4%)	78 (17.3%)	13 (5.5%)	12 (5.9%)
Learning	1490 (42.0%)	209 (48.4%)	328 (41.7%)	203 (40.6%)	100 (33.8%)	11 (12.4%)	165 (51.6%)	67 (27.1%)	277 (61.4%)	60 (25.4%)	70 (34.7%)
Communication	314 (8.8%)	36 (8.3%)	101 (12.8%)	43 (8.6%)	4 (1.4%)	9 (10.1%)	26 (8.1%)	9 (3.6%)	65 (14.4%)	9 (3.8%)	12 (5.9%)
Fine Motor Skills	316 (8.9%)	24 (5.6%)	138 (17.6%)	53 (10.6%)	19 (6.4%)	2 (2.2%)	8 (2.5%)	18 (7.3%)	37 (8.2%)	4 (1.7%)	13 (6.4%)
Acute Respiratory Infection (ARI) prevalence (n, %)	14,704 (28.3%)	1221 (33%)	2876 (29.7%)	2162 (25%)	1137 (31%)	486 (36%)	1347 (29%)	2500 (42%)	1245 (18%)	1023 (24%)	707 (24%)
Diarrhoea Prevalence (n, %)	6739 (13%)	638 (17%)	1815 (18.7%)	821 (10%)	488 (13%)	87 (7%)	402 (9%)	866 (15%)	488 (7%)	686 (16%)	448 (15%)
Fever Prevalence (n, %)	13,357 (26%)	1114 (30%)	2645 (27.3%)	2377 (28%)	919 (25%)	219 (16%)	674 (15%)	2249 (38%)	1436 (20%)	876 (21%)	848 (29%)

Table 2 shows baseline characteristics by disability status. There were more disabled boys than girls (n = 1,952, 55% in boys; n = 1,598, 45% in girls). The mean ages of disabled children and non-disabled children were 3.3 ± 0.6 years and. 3.4 ± 0.6 years respectively. A lower proportion disabled children lived in urban areas than in rural areas (24% vs. 26%, respectively). In most countries, there were fewer disabled children in the top income quintile compared to non-disabled children. The prevalence of ARI, diarrhoea and fever was generally higher in disabled children (28% vs. 33%; 13% vs. 16%; and 26% vs. 29%, respectively), though absolute differences were small, overall.

**Table 2:** Baseline characteristics by disability status, of 51, 901 children from 10 Multiple Indicator Cluster Survey (MICS) countries in sub-Saharan Africa, 2017–2020.

	All countries combined		Central African Republic		Chad		Democratic Republic of Congo		Ghana	
	non-disabled	disabled	non-disabled	disabled	non-disabled	disabled	non-disabled	disabled	non-disabled	disabled
Number of children (n, %)	48,351 (93%)	3550 (7%)	3278 (89%)	423 (11%)	8911 (91.9%)	786 (8.1%)	8098 (94%)	500 (6%)	3382 (92%)	296 (8%)
Female (n, %)	24,415 (51%)	1598 (45%)	1655 (51%)	213 (50%)	4422 (50%)	379 (48%)	4208 (52%)	219 (43.8%)	1728 (51.1%)	136 (45.9%)
Age Mean (SD)	3.4 (0.6)	3.3 (0.6)	3.5 (0.5)	3.4 (0.5)	3.5 (0.5)	3.5 (0.5)	3.5 (0.5)	3.47 (0.5)	3.49 (0.5)	3.49 (0.5)
Urban (n, %)	12,568 (26%)	863 (24%)	1179 (36%)	129 (31%)		154 (19%)	2159 (26.7%)	139 (27.8%)	1373 (40.6%)	98 (33.1%)
Wealth quintile (n,%)										
1 (bottom)	14,160 (29%)	1097 (31%)	1307 (40%)	194 (46%)	2177 (24%)	175 (22%)	2645 (32.7%)	177 (35.4%)	1030 (30.5%)	98 (33.1%)
2	10,688 (22%)	774 (22%)	0 (0%)	0 (0%)	2124 (24%)	189 (24%)	2142 (26.5%)	129 (25.8%)	658 (19.5%)	64 (21.6%)
3	9.691 (20%)	743 (21%)	655 (20%)	77 (18%)	1723 (19%)	173 (22%)	1692 (20.9%)	96 (19.2%)	626 (18.5%)	59 (19.9%)
4	7569 (16%)	532 (15%)	709 (22%)	90 (21%)	1433 (16%)	113 (14%)	1109 (13.7%)	67 (13.4%)	537 (15.9%)	46 (15.5%)
5 (top)	6243 (13%)	404 (11%)	607 (19%)	62 (15%)	1454 (16%)	136 (17%)	510 (6.3%)	31 (6.2%)	531 (15.7%)	29 (9.8%)
Acute Respiratory Infection (ARI) prevalence	13,542 (28%)	1162 (33%)	1029 (31%)	192 (45%)	2621 (29%)	255 (32%)	2018 (24.9%)	144 (28.8%)	1030 (30.5%)	107 (36.1%)
Diarrhoea prevalence	6176 (13%)	563 (16%)	549 (17%)	89 (21%)	1666 (19%)	149 (19%)	758 (9.4%)	63 (12.6%)	442 (13.1%)	46 (15.5%)
Fever prevalence	12,343 (26%)	1014 (29%)	945 (29%)	169 (40%)	2449 (28%)	196 (25%)	2228 (27.5%)	149 (29.8%)	827 (24.5%)	92 (31.1%)

	Lesotho		Madagascar		Malawi		Sierra Leone		The Gambia		Togo	
	Non-disabled	Disabled	Non-disabled	Disabled	Non-disabled	Disabled	Non-disabled	Disabled	Non-disabled	Disabled	Non-disabled	Disabled
Number of children (n, %)	1246 (93%)	89 (7%)	4328 (93%)	320 (7%)	5700 (96%)	247 (4%)	6641 (94%)	451 (6%)	3989 (94%)	236 (6%)	2778 (93%)	202 (7%)
Female (n, %)	632 (50.7%)	35 (39.3%)	2149 (49.7%)	139 (43.4%)	2935 (51.5%)	96 (38.9%)	3369 (50.7%)	203 (45%)	1956 (49%)	98 (41.5%)	1361 (49%)	80 (39.6%)
Age Mean (SD)	3.52 (0.5)	3.56 (0.5)	3.51 (0.5)	3.43 (0.5)	3.49 (0.5)	3.46 (0.5)	3.02 (0.81)	2.66 (0.79)	3.48 (0.5)	3.49 (0.5)	3.02 (0.82)	2.85 (0.8)
Urban (n, %)	307 (24.6%)	24 (27%)	1038 (24.0%)	61 (19.1%)	676 (11.9%)	15 (6.1%)	1935 (29.1%)	129 (28.6%)	1495 (37.5%)	46 (19.5%)	847 (30.5%)	68 (33.7%)
Wealth quintile (n,%)												
1 (bottom)	398 (31.9%)	30 (33.7%)	1373 (31.7%)	91 (28.4%)	1231 (21.6%)	54 (21.9%)	1876 (28.2%)	122 (27.1%)	1428 (35.8%)	111 (47%)	695 (25%)	45 (22.3%)
2	301 (24.2%)	16 (18%)	1028 (23.8%)	83 (25.9%)	1205 (21.1%)	62 (25.1%)	1651 (24.9%)	120 (26.6%)	964 (24.2%)	69 (29.2%)	615 (22.1%)	42 (20.8%)
3	212 (17%)	13 (14.6%)	832 (19.2%)	78 (24.4%)	1181 (20.7%)	57 (23.1%)	1478 (22.3%)	115 (25.5%)	721 (18.1%)	34 (14.4%)	571 (20.6%)	41 (20.3%)
4	183 (14.7%)	16 (18%)	600 (13.9%)	46 (14.4%)	1101 (19.3%)	42 (17%)	916 (13.8%)	59 (13.1%)	511 (12.8%)	9 (3.8%)	470 (16.9%)	44 (21.8%)
5 (top)	152 (12.2%)	14 (15.7%)	495 (11.4%)	22 (6.9%)	982 (17.2%)	32 (13%)	720 (10.8%)	35 (7.8%)	365 (9.2%)	13 (5.5%)	427 (15.4%)	30 (14.9%)
Acute Respiratory Infection (ARI) prevalence	446 (35.8%)	40 (44.9%)	1250 (28.9%)	97 (30.3%)	2370 (41.6%)	130 (52.6%)	1160 (17.5%)	85 (18.8%)	961 (24.1%)	62 (26.3%)	657 (23.7%)	50 (24.8%)
Diarrhoea prevalence	75 (6%)	12 (13.5%)	378 (8.7%)	24 (7.5%)	798 (14%)	68 (27.5%)	451 (6.8%)	37 (8.2%)	640 (16%)	46 (19.5%)	419 (15.1%)	29 (14.4%)
Fever prevalence	194 (15.6%)	25 (28.1%)	628 (14.5%)	46 (14.4%)	2126 (37.3%)	123 (49.8%)	1338 (20.1%)	98 (21.7%)	814 (20.4%)	62 (26.3%)	794 (28.6%)	54 (26.7%)

Table 3 shows odds ratios for having the illness and care seeking behaviours for each illness according to disability status for all countries combined as well as for each country. Table 4 shows odds ratios for the care provider type from whom care is sought for each illness according to disability status for all countries combined; multivariate analyses stratified by country are shown in Supplementary Table S1.

**Table 3:** Unadjusted and adjusted odds ratios for having and seeking care for Acute Respiratory Infection (ARI), diarrhoea, and fever in children from 10 Multiple Indicator Cluster Survey (MICS) countries in sub-Saharan Africa, 2017–2020.

		Country	All countries	Central African Republic	Chad	DRC	Gambia	Ghana	Lesotho	Madagascar	Malawi	Sierra Leone	Togo	Togo
ARI	Having the illness	n	1162	192	255	144	62	107	40	97	130	85	50	50
		OR [95% C.I.]^c^	1.34 [1.17, 1.53]	1.96 [1.44, 2.66]	1.19 [0.93, 1.54]	1.09 [0.64, 1.87]	0.64 [0.40, 1.04]	1.42 [0.89, 2.27]	1.86 [0.88, 3.93]	1.34 [0.89, 2.02]	1.47 [1.02, 2.10]	1.66 [1.11, 2.50]	1.14 [0.61, 2.13]	1.14 [0.61, 2.13]
		aOR [95% C.I.]^b^	1.33 [1.16, 1.52]	1.94 [1.43, 2.64]	1.21 [0.94, 1.56]	1.1 [0.65, 1.88]	0.68 [0.42, 1.10]	1.48 [0.93, 2.37]	2.05 [0.98, 4.28]	1.32 [0.87, 2.00]	1.42 [0.99, 2.04]	1.55 [1.03, 2.33]	1.07 [0.58, 1.97]	1.07 [0.58, 1.97]
	Seeking care for the illness	n	351	52	54	48	a	49	a	25	73	50	a	a
		OR [95% C.I.]^c^	0.90 [0.69, 1.18]	0.51 [0.29, 0.91]	2.20 [1.15, 4.19]	0.71 [0.26, 1.96]	a	1.07 [0.4, 2.85]	a	0.49 [0.24, 0.98]	0.67 [0.35, 1.29]	1.02 [0.37, 2.84]	a	a
		aOR [95% C.I.]^b^	0.90 [0.69, 1.19]	0.50 [0.27, 0.90]	2.05 [1.01, 4.18]	0.74 [0.26, 2.11]	a	1.23 [0.46, 3.30]	a	0.45 [0.22, 0.92]	0.71 [0.36, 1.40]	1.06 [0.39, 2.85]	a	a
Diarrhoea	Having the illness	n	558	89	149	63	46	46	a	31	68	37	29	29
		OR [95% C.I.]^c^	1.30 [1.14, 1.47]	1.50 [1.12, 2.01]	1.09 [0.86, 1.38]	1.3 [0.84, 2.02]	1.03 [0.68, 1.57]	1.07 [0.69, 1.65]	a	0.82 [0.5, 1.35]	2.31 [1.54, 3.47]	1.45 [1.00, 2.1]	1.4 [0.77, 2.53]	1.4 [0.77, 2.53]
		aOR [95% C.I.]^b^	1.27 [1.12, 1.44]	1.44 [1.07, 1.94]	1.07 [0.84, 1.36]	1.29 [0.83, 2.00]	0.98 [0.64, 1.49]	1.07 [0.68, 1.67]	a	0.81 [0.49, 1.32]	2.34 [1.55, 3.52]	1.35 [0.93, 1.95]	1.41 [0.78, 2.55]	1.41 [0.78, 2.55]
	Seeking care for the illness	n	290	39	82	32	29	33	a	a	49	26	a	a
		OR [95% C.I.]^c^	1.07 [0.85, 1.36]	1.02 [0.60, 1.72]	1.88 [1.23, 2.87]	0.73 [0.33, 1.58]	1.27 [0.58, 2.78]	0.57 [0.24, 1.36]	a	a	1.17 [0.56, 2.45]	0.92 [0.41, 2.04]	0.64 [0.22, 1.85]	0.64 [0.22, 1.85]
		aOR [95% C.I.]^b^	1.06 [0.84, 1.34]	0.98 [0.58, 1.67]	1.93 [1.26, 2.95]	0.71 [0.33, 1.55]	1.21 [0.56, 2.59]	0.55 [0.23, 1.33]	a	a	1.18 [0.56, 2.46]	0.76 [0.32, 1.79]	0.63 [0.22, 1.81]	0.63 [0.22, 1.81]
Fever	Having the illness	n	1114	169	196	149	62	92	25	46	123	198	54	54
		OR [95% C.I.]^c^	1.21 [1.08, 1.35]	1.85 [1.45, 2.36]	0.99 [0.79, 1.23]	1.09 [0.77, 1.54]	1.12 [0.77, 1.63]	1.49 [1.07, 2.08]	1.82 [1.02, 3.23]	0.93 [0.64, 1.37]	1.46 [1.04, 2.05]	1.17 [0.89, 1.53]	0.77 [0.52, 1.15]	0.77 [0.52, 1.15]
		aOR [95% C.I.]^b^	1.19 [1.06, 1.33]	1.83 [1.43, 2.34]	0.98 [0.79, 1.22]	1.09 [0.77, 1.54]	1.11 [0.76, 1.6]	1.43 [1.02, 1.99]	1.88 [1.05, 3.36]	0.92 [0.63, 1.35]	1.38 [0.98, 1.96]	1.11 [0.84, 1.46]	0.78 [0.53, 1.15]	0.78 [0.53, 1.15]
	Seeking care for the illness	n	533	66	81	66	34	69	a	26	91	69	31	31
		OR [95% C.I.]^c^	1.06 [0.87, 1.29]	0.91 [0.61, 1.34]	1.47 [1.01, 2.13]	1.42 [0.80, 2.52]	1.02 [0.55, 1.92]	0.89 [0.46, 1.72]	a	0.78 [0.38, 1.57]	1.03 [0.63, 1.68]	0.92 [0.54, 1.56]	0.68 [0.35, 1.33]	0.68 [0.35, 1.33]
		aOR [95% C.I.]^b^	1.07 [0.88, 1.30]	0.91 [0.62, 1.33]	1.48 [1.02, 2.16]	1.5 [0.84, 2.69]	1.06 [0.56, 1.99]	0.90 [0.47, 1.73]	a	0.78 [0.39, 1.55]	1.05 [0.64, 1.71]	0.91 [0.54, 1.54]	0.69 [0.34, 1.38]	0.69 [0.34, 1.38]

a Based on fewer than 25 cases unweighted.

b Adjusted for age, wealth, sex, and location. All countries estimate is also adjusted by country.

c Adjusted for country only.

**Table 4:** Adjusted odds ratios for the care provider type from whom care is sought for Acute Respiratory Infection (ARI), diarrhoea and fever, according to disability status in children from 10 Multiple Indicator Cluster Survey (MICS) countries in sub-Saharan Africa, 2017–2020.

	Care provider type (vs unspecified Health Facility worker) and disability status (vs non-disabled)					
	Unspecified health facility worker		Trained health worker		Non-health professional	
	N	OR Disabled	N	OR [95% CI] Disabled	N	OR [95% CI] Disabled
Country-adjusted only						
ARI care provider	264	1	45	2.43 [1.44, 4.10]	81	1.78 [1.12, 2.84]
Diarrhoea care provider	198	1	42	1.41 [0.81, 2.44]	70	1.09 [0.79, 1.49]
Fever care provider	364	1	81	1.5 [1.04, 2.16]	91	1.13 [0.83, 1.54]
Multivariate (adjusted)^a^						
ARI care provider	264	1	45	2.50 [1.47, 4.25]	81	1.89 [1.19, 2.98]
Diarrhoea care provider	198	1	42	1.35 [0.78, 2.32]	70	1.08 [0.75, 1.56]
Fever care provider	364	1	81	1.49 [1.03, 2.14]	91	1.14 [0.84, 1.54]

a Adjusted for country, wealth, age, location, and sex.

### Acute respiratory infection (ARI)

Disabled children had small overall absolute differences (5%) in ARI prevalence compared to non-disabled children (Table 2). However, this ranged by country, from nearly 14% higher prevalence in disabled children in Central African Republic and Malawi to 1.3% in Sierra Leone. In Togo, this trend was reversed, with non-disabled children had slightly higher prevalence of ARI than disabled children (0.7%). Using logistic regression, in all countries combined, we observed that disabled children had a higher odds of having had an ARI in the past two weeks than non-disabled children (Table 3: aOR = 1.33, 95% C.I. 1.16–1.52). When analysed by country, disabled children had a higher odds of having had an ARI in the past two weeks than non-disabled children in Central African Republic (aOR = 1.94, 95% C.I. 1.43–2.64) and Sierra Leone (aOR = 1.55, 95% C.I. 1.03–2.33). Some estimates were also imprecise, for example, in Lesotho, disabled children had a confidence interval of between 0.98 times lower odds and 4.28 times higher odds of having had a recent ARI compared with non-disabled children (aOR = 2.05, 95% C.I. 0.98–4.28).

When all countries were combined, in children who had an ARI, there was no evidence that caregivers of disabled children had a different odds of seeking care than caregivers of non-disabled children (Table 3: aOR = 0.90, 95% C.I. 0.69–1.19). When analysed by country, caregivers for disabled children in Chad had a higher odds of seeking care for ARI than for non-disabled children (aOR = 2.05, 95% C.I. 1.01–4.18). Conversely, in Central African Republic (aOR = 0.50, 95% C.I. 0.27–0.90) and Madagascar (aOR = 0.45, 95% C.I. 0.22–0.92) caregivers of non-disabled children had lower odds of seeking care for ARI than non-disabled children. Some estimates were imprecise, for example, in Ghana, the odds of seeking care in children with diarrhoea were between 0.46 times lower odds and 3.30 times higher odds for disabled children compared with non-disabled children (aOR = 1.23, 95% C.I. 0.46–3.30).

While the absolute number of caregivers reporting seeing a non-health professional (Table 4: n = 81) was nearly double trained health workers (n = 45), the multinomial logistic regression showed that the odds of seeking care for ARI from a trained health worker compared to from an unspecified health facility worker were higher for caregivers of disabled children than for caregivers of non-disabled children (aOR = 1.76, 95% C.I. 1.25–2.47). The odds of seeking care for ARI from a non-health professional compared to from an unspecified health facility worker was also higher for caregivers of disabled children compared to non-disabled children (aOR = 1.89, 95% C.I. 1.19–2.98).

### Diarrhoea

We also observed similarly small absolute differences in diarrhoea (3%) between disabled and non-disabled children (Table 2). Non-disabled children also had had higher prevalence of diarrhoea and small absolute differences in Madagascar and Togo (by 1.2% and 0.7%, respectively), while disabled children had larger absolute differences in Lesotho (7.5%) and Malawi (13.5%). Using logistic regression models, we found disabled children had a higher odds of diarrhoea in the past two weeks than non-disabled children overall (Table 3: aOR = 1.27, 95% C.I. 1.12–1.44), in Central African Republic (aOR = 1.44, 95% C.I. 1.07–1.94), and in Malawi (aOR = 2.34, 95% C.I. 1.55–3.52). For other countries, we found imprecise estimates, such as in Democratic Republic of Congo where the odds of diarrhoea were between 0.83 times lower to 2.00 times higher in disabled children compared with non-disabled children (aOR = 1.29, 95% C.I. 0.83–2.00).

Furthermore, there was no evidence that caregivers of disabled children had a higher odds of seeking care for diarrhoea than caregivers of non-disabled children overall (Table 3: aOR = 1.06, 95% C.I. 0.84–1.34). When analysed by country, caregivers for disabled children in Chad had a higher odds of seeking care for ARI than for non-disabled children (aOR = 1.93, 95% C.I. 1.26–2.95), while other countries had imprecise estimates, including in The Gambia (aOR = 1.21, 95% C.I. 0.56–2.59) and Malawi (aOR = 1.18, 95% C.I. 0.56–2.46).

There was a similar trend for diarrhoea, in that the absolute number of caregivers seeking care from a non-health professional was higher than a trained health worker (Table 4). However, there was no evidence that the odds of caregivers of disabled children seeking care for diarrhoea from a trained health worker, or from a non-health professional, compared to an unspecified health facility worker, differed compared to caregivers of non-disabled children (Table 4: aOR = 1.35, 95% C.I. 0.78–2.32 and aOR = 1.08, 95% C.I. 0.75–1.56, respectively).

### Fever

Finally, disabled children also had small absolute differences (3%) in having a fever in the past two weeks compared to non-disabled children (Table 2). Malawi had the largest absolute difference (12.5%), while non-disabled children had higher rates of fever and smaller absolute differences in Chad (3%), Togo (1.9%), Madagascar (0.1%). Overall, disabled children had a higher odds of fever in the past two weeks than non-disabled children (Table 3: aOR = 1.19, 95% C.I. 1.06–1.33). When analysing by country, disabled children had a higher odds of fever than non-disabled children in Central African Republic (aOR = 1.83, 95% C.I. 1.43–2.34), Ghana (aOR = 1.43, 95% C.I. 1.02–1.99), and Lesotho (aOR = 1.88, 95% C.I. 1.05–3.36. No association was seen in other countries, although wide confidence intervals resulted in imprecision other countries.

In children who had a fever, the combined estimate for all countries showed no evidence that caregivers of disabled children had a higher odds of seeking care for fever than caregivers of non-disabled children (Table 3: aOR = 1.07, 95% C.I. 0.88–1.30). Similar results were seen when analysed by country, with no evidence that caregivers of disabled children had a higher odds of seeking care for fever than caregivers of non-disabled children, except for Chad (aOR = 1.48, 95% C.I. 1.02–2.16). Most other countries had imprecise estimates imprecise, with wide confidence intervals.

Caregivers of disabled children had a higher odds of seeking care for fever from a trained health worker than from an unspecified health facility worker compared to caregivers of non-disabled children (Table 4: aOR = 1.49, 95% C.I. 1.03–2.14). The odds of seeking care for fever from a non-health professional compared to from an unspecified health facility worker were between 0.95 times lower and 1.54 times greater for caregivers of disabled children compared to non-disabled children (aOR = 1.14, 95% C.I. 0.84–1.54).

## Discussion

In this study of 51,901 children aged 2–4 years from 10 countries in SSA, the odds of having ARI, diarrhoea and fever in the past two weeks were higher in disabled children than in non-disabled children, although the absolute differences were small overall (between 3 and 5%). There was no evidence that caregivers of disabled children had higher or lower odds of seeking care for ARI, diarrhoea, or fever, than caregivers of non-disabled children. However, this trend diverged in Chad, where caregivers of disabled children had higher odds of seeking care for all illness, and in Central African Republic and Madagascar where disabled children had lower odds of seeking care for ARI. Caregivers of disabled children had a higher odds of seeking care for ARI and fever from a trained health worker or non-health professional for ARI than from an unspecified health facility worker, compared to caregivers of non-disabled children, but no association was seen for diarrhoea.

This study examines key indicators for access to health care for disabled children in sub-Saharan Africa—an under-studied population within the SDGs. However, our study has also found some differences with existing literature and conceptions of health inequity for disabled children, including that, in most countries, there were no observed differences between disabled and non-disabled children in care seeking. There are several possible reasons that contribute to the differences observed in this study. First, the sample has notable differences with existing literature on the demographic distribution of disabled children—existing literature suggests disability is more common in older and male children, whereas disabled children were, on average, younger, and more likely to be female. While the analysis was adjusted for sex and age, there are cultural norms around gender that further impact care seeking behaviours (i.e., preference for male children can sometimes result in prioritizing them for limited resources than female children),^18^ treatment, and quality of care. Moreover, the having these illnesses itself are intertwined with disability. For example, some children's disabilities may predispose them to particular illnesses, such as children with neuromuscular conditions who have been found to have higher rates of ARI.^19^ Similarly, disabled people, overall, have been shown to have poorer health status, which may predispose them to more infections.^20^

In addition, disability status is often associated with family poverty,^5^ which can lead disabled children to be exposed to poor water supply, sanitation, and hygiene (WASH)^21^ or have limited health access. While adjusting for wealth may capture some of this interaction, disabled children may also be more exposed to poor sanitation because of lack of accessible WASH facilities,^22^ which could contribute to higher rates of illness observed in this study.^23^ Our results on whether care was sought at different rates are inconclusive, with small numbers and wide confidence intervals. It is possible that differences in health systems, connections to the health system for specialized disability care, or severity of illness may affect care-seeking behaviour and where care is sought, but we were not able to examine this with the available data.

Furthermore, much of the existing literature suggests that disabled children see non-health professionals more often due to cultural associations with disability and stigma.^4^^,^^7^ For example, parents of children with neurodisabilities in Malawi were encouraged by their communities to see traditional healers because of stigma and disability being associated with curses, punishment, and bad luck.^7^^,^^24^ In Senegal, few disabled children knew of specialized services, and instead relied predominantly on traditional doctors or religious healers.^4^ In contrast, our study suggests that caregivers of disabled children sought care for ARI from non-trained health professionals, but also saw trained health professionals more often than caregivers of non-disabled children for ARI and fever. We were not able to analyse where parents sought health care for the disability itself, or the severity of the illness as the data were not available.

This study provides novel analysis on critical indicators for child health for disabled children in the MICS6. While other studies have used proxies for disability in previous rounds of the MICS,^25^ this is the first study to use the CFM to examine these crucial indicators. Since disability disaggregation is routinely missing from SDG monitoring, this study provides critical evidence on differences in having common childhood illnesses and care seeking for disabled and non-disabled children. Furthermore, few studies have examined trends in health providers, making the finding that caregivers usually seek the health advice of trained health workers for ARI and fever, a key contribution to the literature.

However, we were not able to present planned analyses for every county where data were available or conduct the multinomial logistic regression on each individual country due to small numbers (Supplementary Table S1). While we adjusted for country, we were not able to study the prevalence of other diseases, such as malaria, and health systems measures (i.e, routine vaccination programs) that may impact prevalence of illness or care-seeking patterns. In addition, despite the robustness of the MICS data, disabled children are likely under-sampled in the questionnaire due to the lack of cultural and language-specific adjustments to the survey and, generally, different cultural conceptions of disability.^12^ Our results may also be affected by recall bias as exposure and in particular outcomes are self-reported by the caregiver. Furthermore, the sample should be considered in context, since the distribution of baseline characteristics, including age and sex differs to other published data where disability is more common in girls and increases with age.^2^ While this may not substantially impact results, since all estimates were adjusted for age and sex, this may be a limitation of the analysis. Finally, there are other variables, such as family size, broader socio-economic context not captured by wealth, and severity of illness, that affect illness and care-seeking behaviour that we were not able to adjust for. The lack of data on severity, the quality of care, and clinical outcomes after illness limited further exploration of these important factors.

In summary, this study provides evidence of increased common childhood illnesses among disabled children in sub-Saharan Africa, although the absolute differences were small for the overall sample. While it provides no evidence of different rates of care seeking overall, there were differences in where caregivers of disabled children sought care for ARI and fever, compared to non-disabled children in all countries. Country-level variation in these trends complicates these findings, as country-specific trends reinforce or diverge from existing conceptions of access to health care for disabled children in these settings. These findings are among the first to compare illness prevalence and care-seeking behaviour between disabled and non-disabled children in a nationally-representative, high-quality health and development dataset and highlight the complex reality for disabled children. While our finding of small relatively small differences in illnesses may be interpreted positively, the lack of data on severity, care quality, and outcomes make it difficult to conclude that there is sufficient progress on child health indicators for disabled children. Our study highlights the importance of further research on this area and collecting disability disaggregated data to examine inequities and improve disability inclusion in health and development programs. Considering these indicators have been highlighted as part of the SDGs goal to “leave no one behind”, it is concerning that there have been such limited studies on these indicators for disabled children. Having limited research and scarce data on disabled children in sub-Saharan Africa risks mistaking lack of data for the absence of inequity—without fully understanding these differences we will leave disabled children behind and risk missing out on high quality health systems for all.

## Contributors

SR conceptualized the study, accessed and verified the raw data, conducted the analysis, and wrote and edited the manuscript. CD accessed and verified the raw data and supported the data cleaning and analysis and edited the manuscript. EM supported the study design and helped write and edit the manuscript.

## Data sharing statement

Data from UNICEF-supported MICS are publicly available. Our analysis code is also available online.

## Declaration of interests

SR receives consulting fees from the Clinton Health Access Initiative. All other authors have nothing to disclose.
