# Supplementary material for: Association between disability status and health care utilisation for common childhood illnesses in 10 countries in sub-Saharan Africa: a cross-sectional study in the Multiple Indicator Cluster Survey
Source: eClinicalMedicine. 2023 Feb 27;57:101870. doi: 10.1016/j.eclinm.2023.101870 (PMC9989629; doi:10.1016/j.eclinm.2023.101870)
Supplement: Supplementary Table S1 [file mmc1.docx]

**Supplementary Table 1: Adjusted odds ratios for the care provider type from whom care is sought for Acute Respiratory Infection (ARI), diarrhoea and fever, according to disability status by country in children from 10 Multiple Indicator Cluster Survey (MICS) countries in sub-Saharan Africa, 2017-2020**

| **ARI Provider** | | | | | | | | | | | | |
| --- | --- | --- | --- | --- | --- | --- | --- | --- | --- | --- | --- | --- |
|  | **Unadjusted OR** | | | | | | **Adjusted OR (age, sex, wealth)** | | | | | |
| **Country** | **N** | **Unspecified health facility worker** | **N** | **trained health worker OR** | **N** | **non-health professional OR** | **N** | **Unspecified health facility worker** | **N** | **trained health worker OR** | **N** | **non-health professional OR** |
| CAR | 9 | 1.00 | 4 | 0.52 | 13 | 1.35 | 9 | 1.00 | 4 | 0.52 | 13 | 1.40 |
| Chad | 14 | 1.00 | 9 | 2.14 | 12 | 2.08 | 14 | 1.00 | 9 | 1.98 | 12 | 2.01 |
| DRC | 8 | 1.00 | 3 | 0.63 | 6 | 1.22 | 8 | 1.00 | 3 | 0.54 | 6 | 1.05 |
| Gambia | 12 | 1.00 | 1 | 1.33 | 0 | 8.00 | 12 | 1.00 | 1 | 1.16 | 0 | 4.70 |
| Ghana | 21 | 1.00 | 0 | 0.00 | 4 | 4.83 | 21 | 1.00 | 0 | 0.00 | 4 | 4.78 |
| Lesotho | 4 | 1.00 | 0 | 0.00 | 0 | 0.00 | 4 | 1.00 | 0 | 0.00 | 0 | 0.00 |
| Madagascar | 8 | 1.00 | 2 | 0.22 | 6 | 1.60 | 8 | 1.00 | 2 | 0.23 | 6 | 1.74 |
| Malawi | 27 | 1.00 | 6 | 1.78 | 3 | 1.59 | 27 | 1.00 | 6 | 1.68 | 3 | 1.50 |
| Sierra Leone | 23 | 1.00 | 2 | 1.74 | 3 | 1.24 | 23 | 1.00 | 2 | 1.72 | 3 | 1.23 |
| Togo | 2 | 1.00 | 4 | 0.00 | 7 | 6.00 | 2 | 1.00 | 4 | 0.00 | 7 | 9.14 |
| **Diarrhoea Provider** | | | | | | | | | | | | |
|  | **Unadjusted OR** | | | | | | **Adjusted OR (age, sex, wealth)** | | | | | |
| **Country** | **N** | **Unspecified health facility worker** | **N** | **trained health worker OR** | **N** | **non-health professional OR** | **N** | **Unspecified health facility worker** | **N** | **trained health worker OR** | **N** | **non-health professional OR** |
| CAR | 21 | 1.00 | 3 | 0.16 | 14 | 1.13 | 21 | 1.00 | 3 | 0.68 | 14 | 1.19 |
| Chad | 35 | 1.00 | 11 | 1.23 | 35 | 8.57 | 35 | 1.00 | 11 | 1.15 | 35 | 0.81 |
| DRC | 15 | 1.00 | 4 | 2.24 | 12 | 1.78 | 15 | 1.00 | 4 | 2.20 | 12 | 1.76 |
| Gambia | 26 | 1.00 | 2 | 1.11 | 0 | 0.00 | 26 | 1.00 | 2 | 1.26 | 0 | 0.00 |
| Ghana | 31 | 1.00 | 0 | 0.00 | 1 | 0.61 | 31 | 1.00 | 0 | 0.00 | 1 | 0.57 |
| Lesotho | 3 | 1.00 | 0 | 0.00 | 0 | 0.00 | 3 | 1.00 | 0 | 0.00 | 0 | 0.00 |
| Madagascar | 5 | 1.00 | 0 | 0.00 | 4 | 3.18 | 5 | 1.00 | 0 | 0.00 | 4 | 3.43 |
| Malawi | 32 | 1.00 | 7 | 1.75 | 7 | 1.59 | 32 | 1.00 | 7 | 1.71 | 7 | 1.54 |
| Sierra Leone | 24 | 1.00 | 0 | 0.31 | 2 | 0.65 | 24 | 1.00 | 0 | 0.33 | 2 | 0.56 |
| Togo | 6 | 0.77 | 2 | 0.91 | 8 | 1.00 | 6 | 0.78 | 2 | 1.12 | 8 | 1.00 |
| **Fever Provider** | | | | | | | | | | | | |
|  | **Unadjusted OR** | | | | | | **Adjusted OR (age, sex, wealth)** | | | | | |
| **Country** | **N** | **Unspecified health facility worker** | **N** | **trained health worker OR** | **N** | **non-health professional OR** | **N** | **Unspecified health facility worker** | **N** | **trained health worker OR** | **N** | **non-health professional OR** |
| CAR | 27 | 1.00 | 9 | 1.34 | 28 | 1.69 | 27 | 1.00 | 9 | 1.33 | 28 | 1.69 |
| Chad | 36 | 1.00 | 13 | 1.60 | 30 | 1.32 | 36 | 1.00 | 13 | 1.78 | 30 | 1.41 |
| DRC | 44 | 1.00 | 8 | 1.46 | 14 | 0.67 | 44 | 1.00 | 8 | 1.57 | 14 | 0.57 |
| Gambia | 32 | 1.00 | 2 | 0.62 | 1 | 1.58 | 32 | 1.00 | 2 | 0.59 | 1 | 0.88 |
| Ghana | 61 | 1.00 | 2 | 0.28 | 4 | 1.11 | 61 | 1.00 | 2 | 0.28 | 4 | 1.08 |
| Lesotho | 8 | 1.00 | 0 | 0.00 | 0 | 0.00 | 8 | 1.00 | 0 | 0.00 | 0 | 0.00 |
| Madagascar | 15 | 1.00 | 5 | 0.70 | 7 | 0.78 | 15 | 1.00 | 5 | 0.63 | 7 | 0.66 |
| Malawi | 70 | 1.00 | 12 | 2.52 | 4 | 0.40 | 70 | 1.00 | 12 | 2.43 | 4 | 0.38 |
| Sierra Leone | 57 | 1.00 | 4 | 1.29 | 8 | 1.08 | 57 | 1.00 | 4 | 1.31 | 8 | 1.09 |
| Togo | 14 | 0.77 | 8 | 3.78 | 9 | 1.47 | 14 | 0.78 | 8 | 5.00 | 9 | 1.66 |
